# Supplementary material for: Impact and benefit-cost ratio of a program for the management of latent tuberculosis infection among refugees in a region of Canada
Source: PLoS One. 2022 May 19;17(5):e0267781. doi: 10.1371/journal.pone.0267781 (PMC9119458; doi:10.1371/journal.pone.0267781)
Supplement: Attachment — Submitted filename: PLoS One LTBI comments.docx [file pone.0267781.s001.docx]

Dear Editor,

This is with reference to the manuscript entitled "**Impact and benefit-cost ratio of a program for the management of latent tuberculosis infection among refugees in a region of Canada**".  The manuscript written by Jacques Pépin *et al.* is quiet interesting, well designed and planned for further execution. I appreciate to reply some minor comments reply from the authors which are mentioned below:

1. The authors are requested to revise the conclusion part in the **Abstract** portion in the above submitted Proposal.
2. Authors are requested to revise the methodology with detailed work plan describing the study population, duration of study, age of the participants, any standard proforma used for data collection, any specific exclusion criteria involved and statistical analysis used in the study as very limited information’s are available in the current submission.
3. Authors are requested to rewrite Line No. 83 and Line no. 218-220.
4. Authors are requested to add the Statistical analysis in the manuscript including the cost-utility analyses.
5. It would be better if the authors provide the flowchart of the study for better clarification as the above mentioned study is too vast and involved different duration and parameters.
6. The Author Contributions needs to be revised by the authors as these are not according to the Journal guidelines.
7. Lastly, the Authors are requested to please have a final look at the references mentioned as per the Journal guidelines.
